# Outcomes Evaluation of Zero-Profile Devices Compared to Stand-Alone PEEK Cages for the Treatment of Three- and Four-Level Cervical Disc Disease

**DOI:** 10.7759/cureus.775

**Published:** 2016-09-10

**Authors:** Peter C Gerszten, Erin Paschel, Hazem Mashaly, Hatem Sabry, Hasan Jalalod'din, Khaled Saoud

**Affiliations:** 1 Department of Neurological Surgery, University of Pittsburgh Medical Center; 2 Ain Shams University

**Keywords:** anterior cervical discectomy and fusion, spinal fusion, cervical spine, disc herniation, zero-profile

## Abstract

Background: Anterior cervical discectomy and fusion (ACDF) is a well-accepted treatment option for patients with cervical spine disease. Three- and four-level discectomies are known to be associated with a higher complication rate and lower fusion rate than single-level surgery. This study was performed to evaluate and compare zero-profile fixation and stand-alone PEEK cages for three- and four-level ACDF.

Methods: Two cohorts of patients who underwent ACDF for the treatment of three- and four-level disease were compared. Thirty-three patients underwent implantation of zero-profile devices that included titanium screw fixation (Group A). Thirty-five patients underwent implantation of stand-alone PEEK cages without any form of screw fixation (Group B).

Results: In Group A, twenty-seven patients underwent a three-level and six patients a four-level ACDF, with a total of 105 levels. In Group B, thirty patients underwent a three-level and five patients underwent a four-level ACDF, with a total number of 110 levels. In Group A, the mean preoperative visual analog scale score (VAS) for arm pain was 6.4 (range 3-8), and the mean postoperative VAS for arm pain decreased to 2.5 (range 1-7). In group B, the mean preoperative VAS of arm pain was 7.1 (range 3-10), and the mean postoperative VAS of arm pain decreased to 2 (range 0-4). In Group A, four patients (12%) developed dysphagia, and in Group B, three patients (9%) developed dysphagia.

Conclusions: This study found zero-profile instrumentation and PEEK cages to be both safe and effective for patients who underwent three- and four-level ACDF, comparable to reported series using plate devices. Rates of dysphagia for the cohort were much lower than reports using plate devices. Zero-profile segmental fixation devices and PEEK cages may be considered as viable alternatives over plate fixation for patients requiring multi-level anterior cervical fusion surgery.

## Introduction

Cervical spondylosis is a disease characterized by progressive degenerative changes of the cervical intervertebral discs, ligaments, joints, and adjacent vertebrae. Multiple level cervical disc disease, especially three- and four-levels, may present a significant challenge to the spine surgeon [1]. Among the various approaches tailored for surgical management of cervical disc disease including anterior, posterior, or sometimes combined approaches, anterior cervical discectomy and fusion (ACDF) still remains the gold standard surgical approach for cervical spondylotic myelopathy with or without radiculopathy [1-2]. One- and two-level ACDF are commonly performed procedures; however, ACDF for three- and four-level disease are less commonly performed with somewhat limited available clinical outcome data [3].

Many options are available for reconstruction of the discectomy defect after cervical discectomy (the fusion portion of the procedure) including autogenous iliac graft, autologous bone graft, cages (PEEK or titanium) with and without plate, dynamic cages, and an artificial disc. The use of intervertebral cages (especially PEEK) with or without the addition of a cervical plate (stand-alone cage) is now one of most the commonly used methods [1, 4].

Studies have demonstrated the advantages of using an anterior cervical plate with interbody cages and grafts. Anterior cervical plates may increase the rate of fusion, provide better stability, decrease micro-movement of the spine, maintain cervical lordosis (sagittal balance), and reduce the incidence of cage/graft subsidence and dislocation, especially in multiple-levels ACDF. However, the use of an anterior cervical plate has been associated with an increased incidence of postoperative dysphagia, even with the use of low-profile plates. There is also an increased risk of recurrent laryngeal nerve injury, esophageal perforation, and tracheoesophageal fistula as well as an increased incidence of adjacent segment disease when compared to stand-alone cages. Furthermore, plate dislodgement and screw breakage and pullout have been reported [5-9].

Stand-alone cervical cages, in single- and multi-level ACDF, have become more widely adopted by spine surgeons to avoid the potential complications associated with the use of anterior cervical plates. On the other hand, the use of stand-alone cages has been shown in some studies to be associated with a higher incidence of cage subsidence which may lead to loss of cervical lordosis and secondary kyphosis [10-11].

The debate of using either a stand-alone cervical cage versus a cage and anterior cervical plate construct has been an issue of discussion in many publications [4]. Recently, zero-profile devices have been developed with the aim of decreasing the potential complications associated with anterior cervical plating while maintaining the benefits of immediate and solid fixation. Zero-profile interbody fixation devices are designed to be contained entirely within the disc space and do not protrude past the anterior wall of the vertebral body, unlike an anterior cervical plate. This study was undertaken to evaluate and compare two groups of patients treated for multi-level (three- and four-levels) cervical disc disease with the use of either a stand-alone zero-profile device or a stand-alone PEEK cage.

## Materials and methods

### Patients

Patients were recruited from two academic medical institutions: The University of Pittsburgh Medical Center, Pittsburgh, USA and Ain Shams University Hospitals, Cairo, Egypt. The study compared two cohorts of patients (A and B) who underwent ACDF for the treatment of multilevel (three- and four-levels) symptomatic cervical disc disease. Group A included 33 patients in whom interbody fusion was performed with zero-profile devices. Devices implanted included either the Optio-C (Zimmer Spine, Minneapolis, MN) or Stalif C (Sentinel Spine, New York, NY). Patients in this group were treated between January 2013 and April 2015 with a follow-up of at least six months. Group B included 35 patients in which stand-alone PEEK cages were utilized for cervical interbody fusion. Patients in this group were treated between January 2009 and October 2013 with follow-up of at least six months. The patients agreed to participate and were explained the nature and objectives of this study, and informed consent was formally obtained. No reference to the patients' identities were made at any stage during data analysis or in the report.

Inclusion criteria for patients were identical for both groups. They included: 1) persistent neck pain, signs and symptoms of cervical radiculopathy/cervical spondylotic myelopathy with failure to respond to at least three months of conservative treatment, and 2) the presence of three- or four-levels of cervical disc disease as evidenced by imaging. Patients with significant cervical spondylotic myelopathy who were believed not be candidates for non-surgical therapies were offered surgery directly. The exclusion criteria were: 1) cervical pathologies other than cervical disc disease, such as infections or ossification of the posterior longitudinal ligament, 2) cases with less than three-levels of cervical disc disease, and 3) a need for both anterior and posterior approaches.

Medical records were reviewed to identify demographic data, comorbidities, clinical presentation, and visual analog score (VAS) of both neck and arm pain and both preoperative and postoperative conditions. The perioperative and intraoperative data such as operative level, blood loss, complications, and length of hospital stay were also reviewed. Preoperative imaging studies including MR imaging and plain radiographs of the cervical spine were evaluated. Postoperative radiographs in the immediate postoperative period and three months after surgery were examined to identify cage subsidence. In this study, subsidence was defined as a decrease in the disc space height by more than 2 mm on lateral x-ray film between the immediate and three-months’ postoperative x-ray imaging. 

### Surgical technique

A standard anterior Smith-Robinson approach was performed in all cases. A Casper retractor was used to allow for a slight distraction followed by a microdiscectomy, and then removal of the posterior cervical osteophyte was carried out by using a high-speed drill and Kerrison rongeurs. Adequate decompression of the neural elements was then ensured by opening/excision of the posterior longitudinal ligament in all cases. During preparation of the fusion bed, great care was taken to avoid excess injury to the cartilaginous end plate and exposure of the subchondral bone. Interbody fusion was then performed in group A with the zero-profile devices and in group B with stand-alone PEEK cages filled with an allograft bone graft substitute. An external orthosis using a hard cervical collar with a chin support for a four-to-six-week period was prescribed for all patients in group B only.

## Results

### Patient population

Group A included thirty-three patients with a mean age of 60 years (range 41-75 years). There were twenty-one males and twelve females. Six patients (18%) had diabetes mellitus, fourteen patients (42%) were hypertensive, seven patients (21%) had symptomatic coronary artery disease, and five (15%) were active smokers at the time of surgery. Eighteen patients (54%) presented with a primary diagnosis of radiculopathy, ten patients (30%) presented with myelopathy, and three (9%) patients with both radiculopathy and myelopathy. Two patients presented with only persistent neck pain. Twenty-five patients (75%) had neck pain at the time of surgery.

Group B included thirty-five patients with a mean age of 52 years (range 42-70 years). There were thirty males and five females. Nine patients (25%) were diabetic, twenty-six patients (45%) had hypertension, and twelve patients (34%) were actively smoking at the time of surgery. All patients in this group had radiculopathy, and six patients (17%) had both radiculopathy and myelopathy. Thirty-three patients (94%) had neck pain at the time of surgery.

### Operative and perioperative data

**Figure 1:**
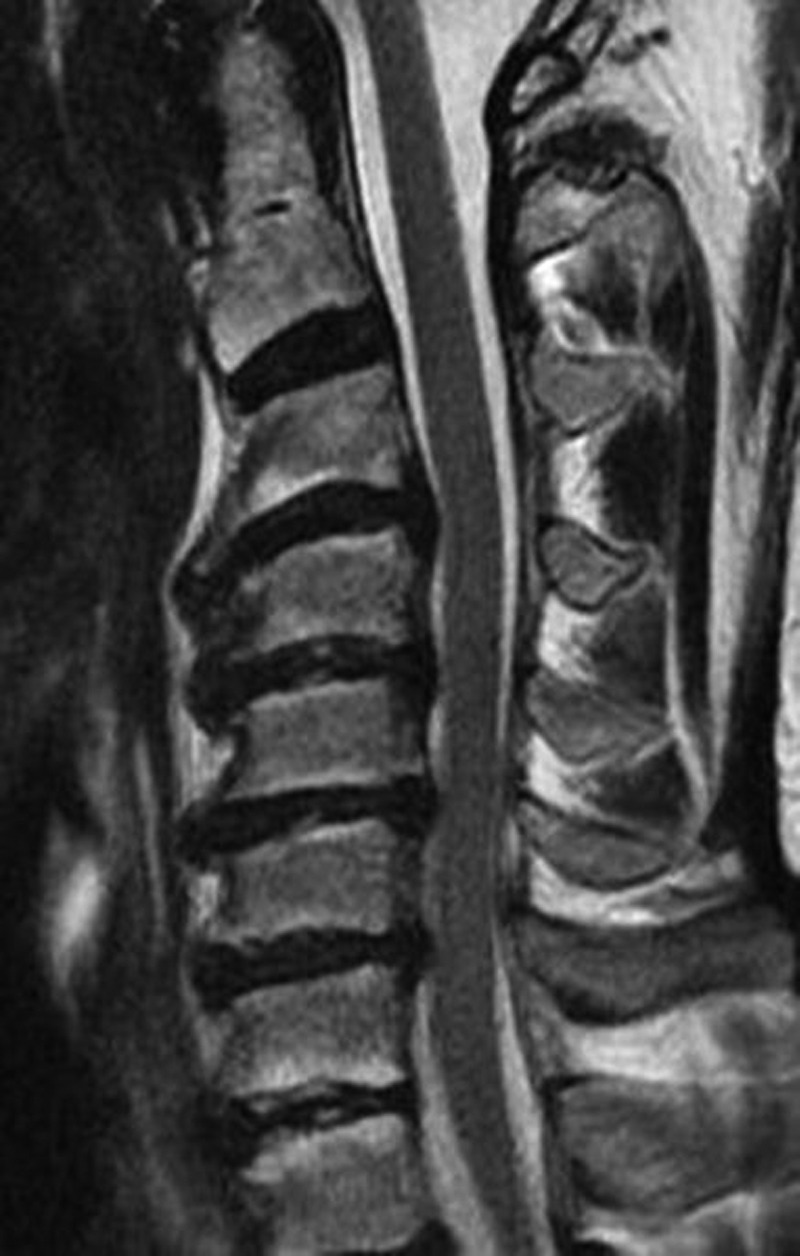
Case Example of a Four-Level ACDF Using the Optio-C Implant A 65-year-old man presented with progressive cervical spondylotic myelopathy with a sagittal T2 weighted MRI demonstrating four levels of cervical stenosis.

In Group A, all patients received interbody fusion with a zero-profile device, twelve patients with Stalif C and twenty-one patients with Optio-C. Twenty-seven patients underwent a three-level ACDF and six patients a four-level ACDF, with a total of 105 levels. Figure 1 demonstrates preoperative imaging of a patient electing to undergo a four level ACDF.

**Figure 2:**
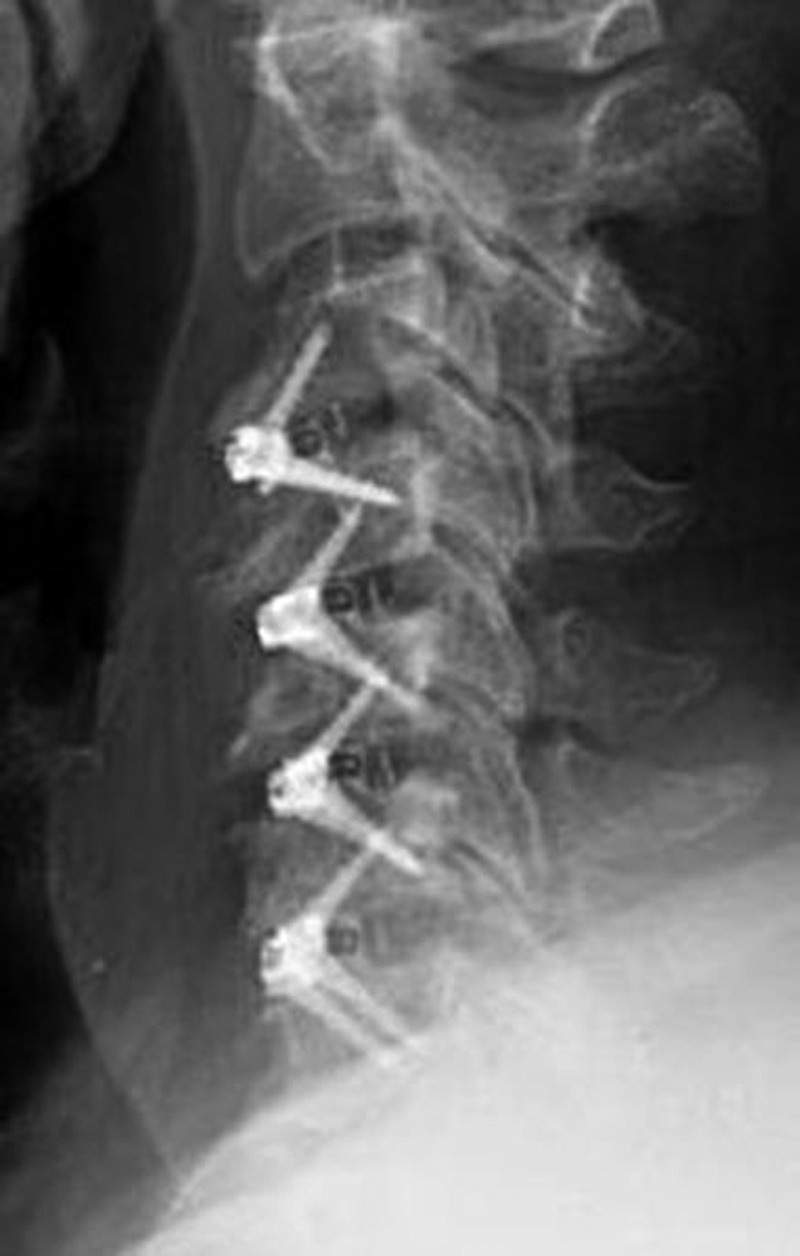
Case Example of a Four-Level ACDF Using the Optio-C Implant Sagittal radiograph at three months after surgery demonstrating preservation of disc heights.

**Figure 3:**
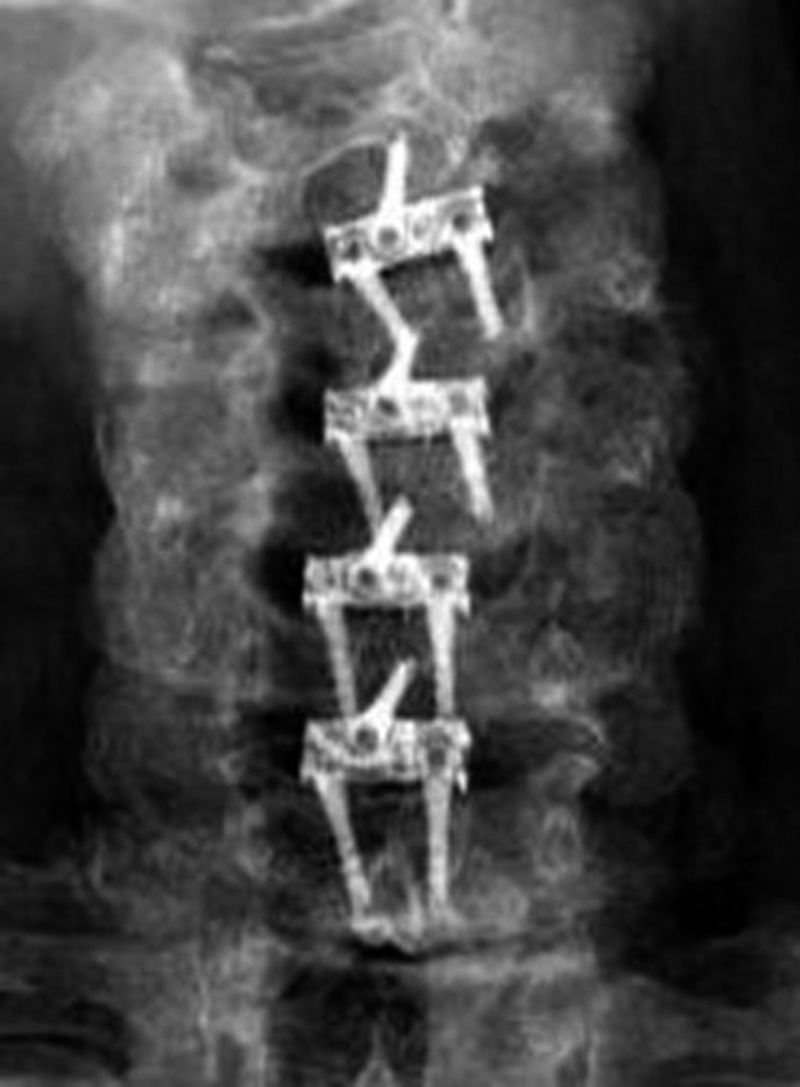
Case Example of a Four-Level ACDF Using the Optio-C Implant AP radiograph at three months after surgery demonstrating preservation of disc heights.

Figures 2-3 demonstrate the postoperative imaging. The most commonly instrumented levels were the C4-C5 level (n= 33) and the C5-C6 level (n=32), followed by the C6-C7 level (n=25), then the C3-C4 level (n=14), and only one cage at the C2-C3 level.

**Figure 4:**
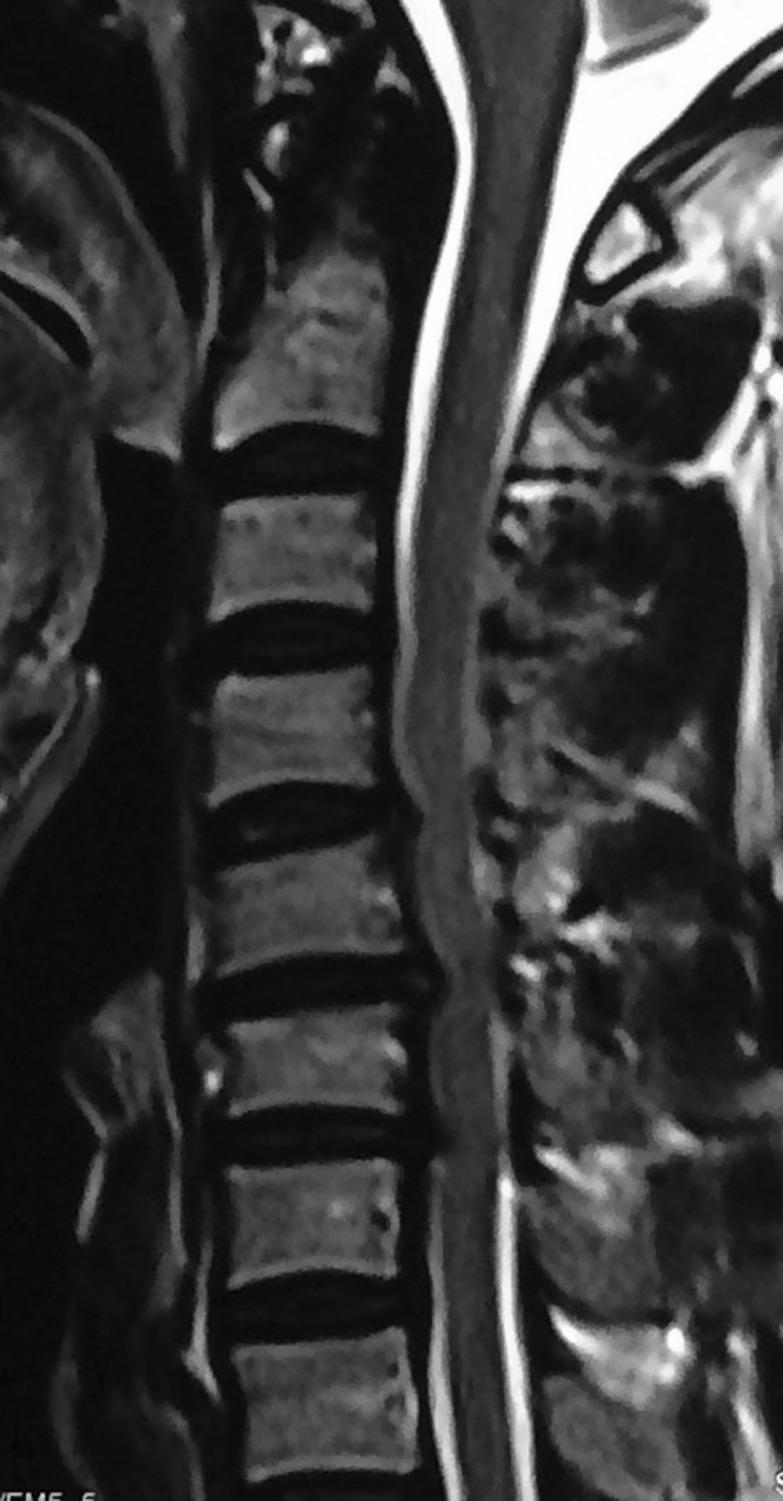
Case Example of a Three-Level ACDF Using Stand-Alone PEEK Cages A 52-year-old man presented with complaints of neck pain, radiculopathy, and cervical spondylotic myelopathy with a sagittal T2 weighted MRI demonstrating three cervical disc herniations.

In Group B, all patients received an interbody fusion using a stand-alone PEEK cage. Thirty patients were operated on for three-levels and five patients were operated upon for four-levels, with a total number of 110 levels. Figure 4 demonstrates preoperative imaging of a patient electing to undergo a three level ACDF.

**Figure 5:**
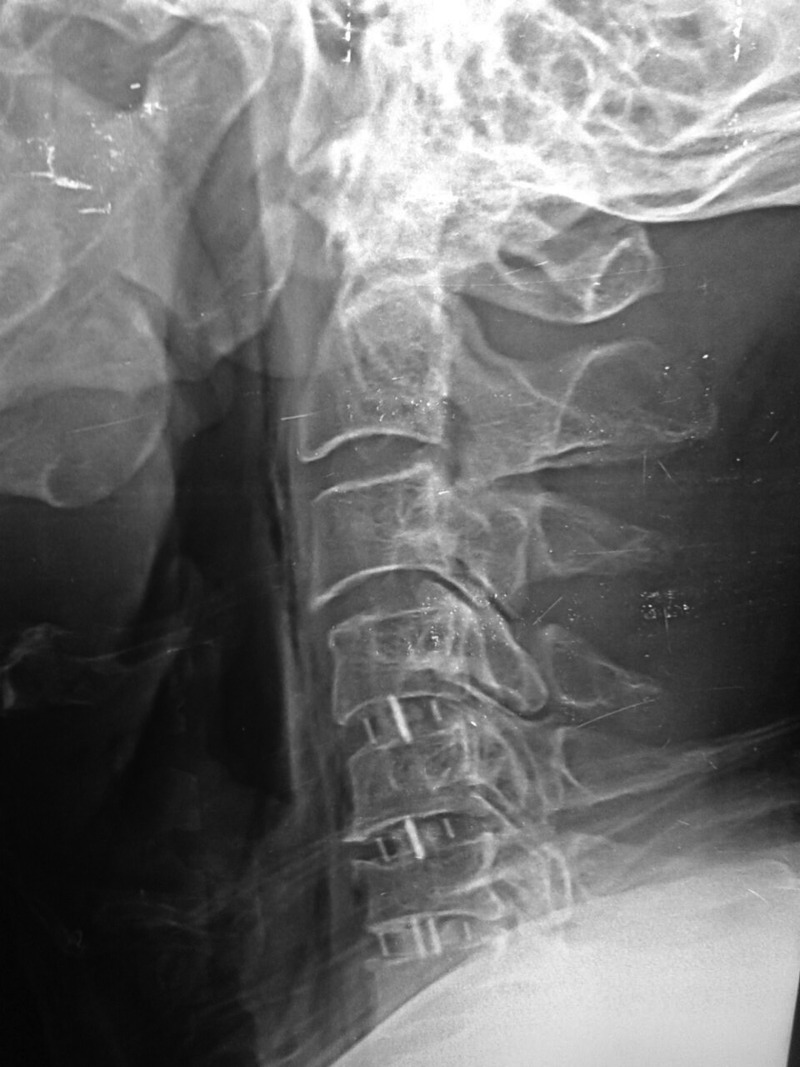
Case Example of a Three-Level ACDF Using Stand-Alone PEEK Cages Sagittal radiograph at three months after surgery demonstrating hardware placement and preservation of disc space heights.

**Figure 6:**
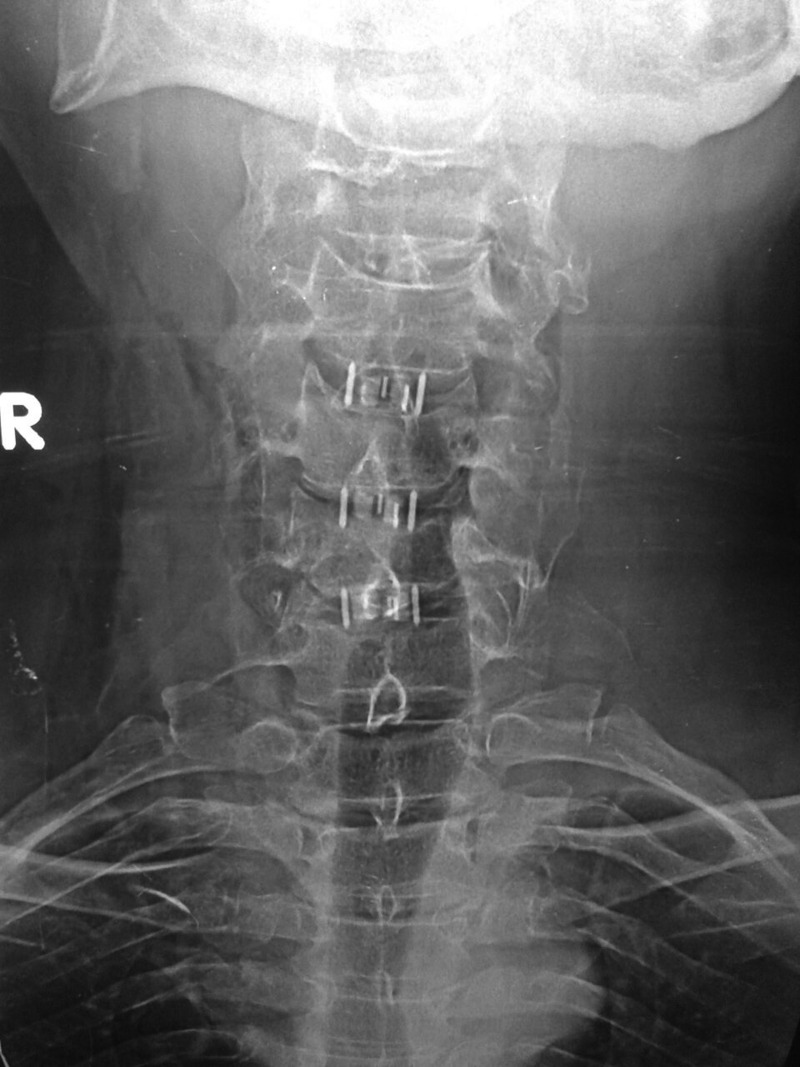
Case Example of a Three-Level ACDF Using Stand-Alone PEEK Cages AP radiograph at three months after surgery demonstrating hardware placement and preservation of disc space heights.

Figures 5-6 demonstrate the postoperative imaging. The most commonly instrumented levels were the C4-C5 and C5-C6 levels (n=35 for each), followed by the C3-C4 level (n=22), and then the C6-C7 level (n=18).

There were no intraoperative complications in either group. The blood loss in both groups was minimal for all cases. The average hospital stay in group A was 1.4 days, while in group B it was 2.3 days. No infections occurred in either group.

### Clinical outcomes

In Group A, the mean preoperative VAS of arm pain was 6.4 (range 3-8), and the mean postoperative VAS of arm pain decreased to 2.5 (range 1-7). The mean preoperative VAS for neck pain was 6.8 (range 5-9), and the mean postoperative VAS for neck pain decreased to 1.7 (range 1-3).

In Group B the mean preoperative VAS of arm pain was 7.1 (range 3-10), and the mean postoperative VAS of arm pain decreased to 2.0 (range 0-4). The mean preoperative VAS for neck pain was 5.6 (range 2-10), and the mean postoperative VAS for neck pain decreased to 3.0 (range 0-6).

### Dysphagia

The severity of dysphagia was graded as none, mild, moderate, or severe as defined according to the Bazaz scoring system as seen in Table 1 on the first postoperative day, first month, three months, and six months postoperatively [12].

**Table 1:** Bazaz Scoring System for Dysphagia

Symptom Severity	Liquid Food	Solid Food
None	None	None
Mild	None	Rare
Moderate	None or rare	Occasional with specific food
Severe	None or rare	Frequent with majority of food

In Group A, four patients (12%) developed dysphagia in the postoperative period; three of whom had mild dysphagia that resolved within the first month of follow-up, and one patient suffered moderate dysphagia which resolved at the three-month follow-up. None of the patients had dysphagia six months after the surgery. In Group B, three patients (9%) developed mild dysphagia in the postoperative period which resolved within the first month following surgery.

### Subsidence

In Group A, five patients (15%) developed cage subsidence, with a total of 7 cages (7% of the total number of cages). All of the cases of subsidence were asymptomatic and were discovered only on routine postoperative imaging.

In Group B, six patients developed subsidence (14%), with a total of 10 cages (9% of the total number of cages). Two of these patients had persistent neck pain with recurrence of radiculopathy which was managed by revision surgery with corpectomy and plate fixation, while four patients had documentation of asymptomatic subsidence.

### Adjacent level degeneration

None of the patients in Group A developed adjacent segment degeneration during the follow-up period. In comparison, two patients in Group B (6%) developed adjacent level degeneration. One case was asymptomatic, while the other patient presented with symptomatic C6-C7 cervical disc disease that ultimately required surgical intervention.

## Discussion

Multi-level disc disease (three- and four-levels) of the cervical spine represents a challenging problem for surgical correction. Although a variety of anterior, posterior, and combined approaches with and without instrumentation have been advocated for multi-level cervical disc disease, the anterior approach still represents the preferable approach in many cases as it allows for direct decompression of the spinal cord and nerve roots as well as achieving solid fusion [1, 13].

PEEK cages have several advantages over autograft and cadaveric allograft implants. The PEEK cage has a hard frame to resist the cervical loading and is more rigid than an iliac bone graft. In laboratory studies, a PEEK cage has good stiffness in compression and rotation tests. It is also safe in regard to histocompatibility. The PEEK cage is wedge-shaped, something which facilitates the creation of a lordotic spinal curvature when placed into the distracted disc space [14].

The use of stand-alone cages in ACDF, although technically easier and possibly avoiding the potential risks of using cervical plates, has been reported in some studies to have potential downsides, including lower stability in extension, a higher incidence of disc space subsidence leading to late kyphosis, and higher rates of pseudoarthrosis. The primary reason for the use of additional plate fixation in addition to cage-assisted ACDF is the high-cage subsidence rate in studies using cages alone without plating. Song et al., found a higher incidence of subsidence in a stand-alone cage technique (32%) as compared to cage and plate technique (10%). Recent data suggest that subsidence usually happens within the first three months after surgery. Some authors suggest that disc space subsidence is actually a natural/physiologic process during fusion [4, 15-16].

Cho et al., studied 180 consecutive cases of multilevel ACDF with three different fusion techniques. They found that PEEK cages and autogenous iliac crest graft with anterior cervical plate are both satisfactory methods for interbody fusion in cases of multilevel ACDF. The complication rate was actually lower in the PEEK group [14]. Many studies, both laboratory and clinical, have highlighted the necessity of additional support to cervical cages by using an anterior cervical plate to prevent excess movement in flexion and extension and also show that using an anterior cervical plate increases rates of arthrodesis [2].

The augmentation of a cervical cage with an anterior cervical plate has numerous advantages. It can decrease the micro-movements of the cervical spine, achieve higher fusion rates, and restore the normal (physiological) cervical spine lordosis. It may also lead to better axial pain relief and lower reoperation rates. However, using long anterior cervical plates (especially in multi-level ACDF) is not without risk and can lead to various complications such as screw breakage, screw pullout, an increased incidence of postoperative dysphagia, and injury of the recurrent laryngeal nerves or even injury of the esophagus [13, 17-18].

The proven benefits of an anterior cervical plate in improving the outcomes of ACDF and at the same time the documented complications, especially postoperative dysphagia, have led to the development of low-profile and subsequently zero-profile cervical implants (e.g., arthrodesis devices) [9]. The design of zero-profile devices combines both an interbody cage that is necessary for stability, restoration of disc height and enhancement of fusion, along with an anterior “plate” that provides further stability of the spine. The design of the device does not extend beyond the anterior edge of the vertebral body and accordingly minimizes contact with adjacent levels and prevertebral soft tissues such as the esophagus. Scholz et al., found that zero-profile implants can provide a biomechanical stability which is comparable to that of the standard plate and cage technique [19-20].

Stein et al., in their anatomical study, found no statistically significant difference in the range of motion between zero-profile cages and cages augmented by an anterior cervical plate in all directions of motion [9]. Another cadaveric study also found no significant difference in stability between zero-profile cages alone and cages supplemented by an anterior plate [21].

Following ACDF with an anterior plate, the rates of persistent dysphagia (defined as more than three months) range between 12% and 35%. The pathophysiological mechanism of dysphagia is still not entirely clear. One of the theories is the direct contact of the cervical plate with the esophagus which might impinge or irritate the esophagus [17, 22-23]. In their study, Lee et al., showed that decreasing the cervical plate thickness from 2.6 mm to 1.6 mm was associated with a reduction of the rate of dysphagia from 22% to 14% at six months [12]. Because the zero-profile implant is completely contained within the intervertebral disc space and does not protrude past the anterior body of the vertebrae, it avoids direct contact with and irritation of the esophagus and therefore may lead to a lower incidence of post-operative dysphagia [23-24].

The incidence of symptomatic adjacent level disease following ACDF is approximately 19% at ten years. Approximately 7% to 15% of patients who undergo an ACDF will require another cervical discectomy surgery, which is typically more challenging and carries a higher risk of complications. The use of zero-profile cages in such cases, especially if a cervical plate were used in the initial surgery, offers some advantages such as minimal dissection of the prior surgical level. Its use also obviates the need to remove a previously placed plate and minimal retraction, which in turn may reduce operative time and risk of postoperative dysphagia [19, 25].

Several studies have compared the clinical outcomes between stand-alone cages and cages supplemented by an anterior cervical plate for cervical disc disease. These studies have reported similar clinical outcomes between the two devices. Other studies have compared the outcomes between zero-profile devices with an anterior cervical plate and a cage. Shin et al., compared three groups of patients: (A) zero-profile devices, (B) stand-alone PEEK cages, and (C) cervical anterior plate with autologous bone graft for single-level cervical disc disease. They reported no significant clinical difference between the three groups. However, they reported a similar incidence of dysphagia in group A and group B, and a higher incidence of dysphagia in group C--the group with cervical plates implanted [11].

De la Garza-Ramos et al., recently published their data on long-term follow-up of three- and four-levels ACDF. They reported higher rates of complications in the four-levels group than with the three-levels group, with dysphagia being one of the most common complications. The incidence of dysphagia was 30% in the four-levels ACDF and 12.7% in the three-levels ACDF. They also reported adjacent level degeneration requiring surgery in 15.6% in the three-level group and 3.9% in the four-level group [3].

The current study demonstrates the safety as well as the clinical efficacy of both zero-profile devices and stand-alone PEEK cages in the surgical treatment of three- and four-level cervical disc disease with satisfactory radiological outcomes. The study compared the clinical and radiological outcomes between zero-profile devices (Group A) and stand-alone PEEK cages (Group B) in multi-level (three- and four-levels) cervical disc disease, with a special focus on neck pain, arm pain, the occurrence of dysphagia, and disc-space subsidence. In this study, the mean preoperative VAS for arm pain in group A decreased from 6.4 to 2.5 post-surgery and in Group B from 7.1 to 2 post-surgery. The mean preoperative VAS for neck pain in Group A decreased from 6.8 to 1.7 post-surgery and in Group B from 5.6 to 3 post-surgery.

In this study, the incidence of dysphagia in the zero-profile and PEEK groups was 12% and 9%, respectively. However, the incidence of dysphagia in both groups was lower than reported incidences with the use of anterior cervical plate constructs [18, 21-22, 26-28]. In the current study, the zero-profile device group showed a 12% incidence of dysphagia, with three patients experiencing mild dysphagia that resolved in the first month following surgery and one patient having moderate dysphagia that resolved three months following surgery. There were no cases of persistent (chronic) dysphagia in the zero-profile group. The stand-alone PEEK group showed a 9% incidence of dysphagia; all cases were mild and disappeared within the first month following surgery.

The risk factors associated with implant subsidence have been reported and include obesity, smoking, poor bone mineral density as well as surgery-related factors such as the anteroposterior diameter of the cage implant and excess intraoperative distraction. However, subsidence does not always lead to a poor clinical outcome or recurrence of symptoms. In fact, many cases remain asymptomatic and ultimately demonstrate a solid radiographic fusion [25, 29-30].

In the zero-profile group, five patients (15%) developed radiographic cage subsidence with a total number of 7 cages (7% of operated levels). All of these cases were asymptomatic and required no further intervention. In the PEEK group, six patients (14%) developed radiographic cage subsidence, with a total number of 10 cages (9% of operated levels). However, unlike in the zero-profile group, two PEEK patients became symptomatic and required further surgery.

Zero-profile devices and stand-alone PEEK cages are both safe and straight forward to insert. The clinical outcomes of both groups are comparable to the outcomes of studies reporting the use of anterior cervical plate constructs, with lower incidence of dysphagia. The procedure of the implantation of zero-profile devices is similar to that of the stand-alone PEEK cages. The screws of the plate anchored to the zero-profile device can be easily inserted through predetermined guided trajectories as opposed to the anterior cervical plate construct placement where the surgeon must manipulate the angles of the screws and choose the appropriate length of the plate. This screw insertion can frequently be both difficult and challenging, especially with a three- and four-level ACDF operation.

## Conclusions

Three- and four-level ACDFs remain challenging cases, with high complication rates and lower clinical outcomes compared two single and two-level ACDF. This study found zero-profile instrumentation and PEEK cages to be both safe and effective for patients who underwent three- and four-level ACDF, comparable to reports using plates. The rates of dysphagia for the entire cohort were indeed lower than in previously reported series using plate fixation devices for three- and four-level ACDF. PEEK cages alone compared to zero-profile devices were found to have a slightly higher incidence of both symptomatic subsidence as well as adjacent level degeneration. Zero-profile segmental fixation devices and PEEK cages may be considered over plates for patients requiring multi-level anterior cervical fusion surgery.

## References

[1] Shousha M, Ezzati A, Boehm H (2012). Four-level anterior cervical discectomies and cage-augmented fusion with and without fixation. Eur Spine J.

[2] Qi M, Chen H, Liu Y, Zhang Y, Liang L, Yuan Yuan, W W (2013). The use of a zero-profile device compared with an anterior plate and cage in the treatment of patients with symptomatic cervical spondylosis: A preliminary clinical investigation. Bone Joint J.

[3] De la Garza-Ramos R, Xu R, Ramhmdani S (2016). A. Long-term clinical outcomes following 3- and 4-level anterior cervical discectomy and fusion. J Neurosurg Spine.

[4] Pereira EA, Chari A, Hempenstall J, Leach JC, Chandran H, Cadoux-Hudson TA (2016). Anterior cervical discectomy plus intervertebral polyetheretherketone cage fusion over three and four levels without plating is safe and effective long-term. J Clin Neurosci.

[5] Barbagallo GM, Romano D, Certo F, Milone P, Albanese V (2013). A new zero-profile cage-plate device for single and multilevel ACDF. A single institution series with four years maximum follow-up and review of the literature on zero-profile devices. Eur Spine J.

[6] Fountas KN, Kapsalaki EZ, Nikolakakos LG (2007). Anterior cervical discectomy and fusion associated complications. Spine.

[7] Kaiser MG, Haid RW Jr, Subach BR, Barnes B, Rodts GE Jr (2002). Anterior cervical plating enhances arthrodesis after discectomy and fusion with cortical allograft. Neurosurgery.

[8] Silber JS, Anderson DG, Daffner SD (2003). Donor site morbidity after anterior iliac crest bone harvest for single-level anterior cervical discectomy and fusion. Spine.

[9] Stein MI, Nayak AN, Gaskins RB 3rd, Cabezas AF, Santoni BG, Castellvi Castellvi, AE AE (2014). Biomechanics of an integrated interbody device versus ACDF anterior locking plate in a single-level cervical spine fusion construct. Spine J.

[10] Moon HJ, Kim JH, Kim JH, Kwon TH, Chung HS, Park YK (2011). The effects of anterior cervical discectomy and fusion with stand-alone cages at two contiguous levels on cervical alignment and outcomes. Acta Neurochir (Wien).

[11] Shin JS, Oh SH, Cho PG (2014). Surgical outcome of a zero-profile device comparing with stand-alone cage and anterior cervical plate with iliac bone graft in the anterior cervical discectomy and fusion. Korean J Spine.

[12] Lee MJ, Bazaz R, Furey CG, Yoo J (2005). Influence of anterior cervical plate design on dysphagia: a 2-year prospective longitudinal follow-up study. J Spinal Disord Tech.

[13] Demircan MN, Kutlay AM, Colak A (2007). Multilevel cervical fusion without plates, screws or autogenous iliac crest bone graft. J Clin Neurosci.

[14] Cho DY, Lee WY, Sheu PC (2014). Treatment of multilevel cervical fusion with cages. Surg Neurol.

[15] Börm W, Seitz K (2004). Use of cervical stand-alone cages. Eur Spine J.

[16] Song KJ, Yoon SJ, Lee KB (2012). Three- and four-level anterior cervical discectomy and fusion with a PEEK cage and plate construct. Eur Spine J.

[17] Miao J, Shen Y, Kuang Y (2013). Early follow-up outcomes of a new zero-profile implant used in anterior cervical discectomy and fusion. J Spinal Disord Tech.

[18] Zhou J, Li X, Dong J (2011). Three-level anterior cervical discectomy and fusion with self-locking stand-alone polyetheretherketone cages. J Clin Neurosci.

[19] Healy AT1, Sundar SJ, Cardenas RJ (2014). Zero-profile hybrid fusion construct versus 2-level plate fixation to treat adjacent-level disease in the cervical spine. J Neurosurg Spine.

[20] Scholz M, Schnake KJ, Pingel A, Hoffmann R, Kandziora F (2011). A new zero-profile implant for stand-alone anterior cervical interbody fusion. Clin Orthop Relat Res.

[21] Njoku I Jr, Alimi M, Leng LZ (2014). Anterior cervical discectomy and fusion with a zero-profile integrated plate and spacer device: a clinical and radiological study: clinical article. J Neurosurg Spine.

[22] Hofstetter CP, Kesavabhotla K, Boockvar JA (2015). Zero-profile anchored spacer reduces rate of dysphagia compared with ACDF with anterior plating. J Spinal Disord Tech.

[23] Wang Z, Jiang W, Li X (2015). The application of zero-profile anchored spacer in anterior cervical discectomy and fusion. Eur Spine J.

[24] Wang ZD, Zhu RF, Yang HL (2014). The application of a zero-profile implant in anterior cervical discectomy and fusion. J Clin Neurosci.

[25] Hilibrand AS, Carlson GD, Palumbo MA, Jones PK, Bohlman HH (1999). Radiculopathy and myelopathy at segments adjacent to the site of a previous anterior cervical arthrodesis. J Bone Joint Surg Am.

[26] Bazaz R, Lee MJ, Yoo JU (2002). Incidence of dysphagia after anterior cervical spine surgery: a prospective study. Spine.

[27] Riley LH 3rd, Skolasky RL, Albert TJ, Vaccaro AR, Heller JG (2005). Dysphagia after anterior cervical decompression and fusion: prevalence and risk factors from a longitudinal cohort study. Spine.

[28] Smith-Hammond CA, New KC, Pietrobon R, Curtis DJ, Scharver CH, Turner DA (2004). Prospective analysis of incidence and risk factors of dysphagia in spine surgery patients: comparison of anterior cervical, posterior cervical, and lumbar procedures. Spine.

[29] Wu WJ, Jiang LS, Liang Y, Dai LY (2012). Cage subsidence does not, but cervical lordosis improvement does affect the long-term results of anterior cervical fusion with stand-alone cage for degenerative cervical disc disease: a retrospective study. Eur Spine J.

[30] Yang JJ, Yu CH, Chang BS, Yeom JS, Lee JH, Lee CK (2011). Subsidence and nonunion after anterior cervical interbody fusion using a stand-alone polyetheretherketone (PEEK) cage. Clin Orthop Surg.

